# Learning Communities in Medical Education: Impact, Integration, and Best Practices

**DOI:** 10.1007/s40670-026-02776-7

**Published:** 2026-06-03

**Authors:** Calvin L. Gruss, William B. Cutrer, Amy E. Fleming, Kendra Parekh, Elizabeth Ann Yakes

**Affiliations:** 1 Department of Anesthesiology, Vanderbilt University School of Medicine, Nashville, TN USA; 2 Department of Pediatrics, Vanderbilt University School of Medicine, Nashville, TN USA; 3 Department of Emergency Medicine, Vanderbilt University School of Medicine, Nashville, TN USA; 4 Department of Medicine, Vanderbilt University School of Medicine, Nashville, TN USA

**Keywords:** Learning communities, Medical education, Coaching, Wellness, Clinical skills, Professional identity formation

## Abstract

Learning communities (LCs) are longitudinal, relationship-centered structures that foster belonging, mentorship, and collaboration across diverse educational settings. This monograph focuses on LCs in undergraduate medical education (UME), summarizing their structures and models; evidence for effects on the learning environment, advising, well-being, and clinical skills; and practical strategies for implementation. We outline common elements of learning communities across the UME spectrum, including protected small-group time, curricular integration, and structured reflection. We then present the Vanderbilt Colleges as a case study demonstrating these design principles in practice. A checklist, pitfalls, and research priorities are offered to support adoption and continuous improvement.

## Introduction and Rationale

Learning communities (LCs) in medical education are purposefully designed, longitudinal groups that pair small cohorts of students with dedicated faculty to promote learning, mentoring, and support over time [1]. In practice, LCs commonly combine longitudinal mentoring and academic advising with structured wellness supports and skills development in small groups [2, 3]. National survey data indicate that roughly half of U.S. medical schools have formal LC structures, with continued growth over time [3]. Hafferty and Watson describe this trend as a “socio-structural” shift in medical training, analogous to the collegiate house system, aimed at strengthening community and belonging within the larger institution [4].

Learning communities (LCs) consist of longitudinal groups of teachers, mentors, and peers who engage in reflection, feedback, skills development, and exploration of professional values. These activities and structures mirror the theoretical framework of Communities of Practice (CoPs) originally described by Lave and Wenger [5], which conceptualizes learning as a social process occurring through participation in a community with shared goals and practices. This framework was later extended to medical education by Cruess et al. [6] to describe the social nature of learning and professional identity formation (PIF). LCs function as effective CoPs in that learners share a common purpose (domain), build relationships in community with peers and faculty (community), and acquire the skills and behaviors required to competently practice medicine (practice) [7]. Within these CoPs, LC members, including peers and faculty, facilitate students’ developing sense of belonging, mattering, and competence through LC activities, thereby influencing PIF [8].

## Structure and Longitudinal Integration

LCs differ in the degree to which they deliver formal curriculum versus focusing on advising and/or mentoring. Many also incorporate longitudinal clinical-skills instruction within small-group settings, linking early professional development to patient-care competencies. Nevertheless, longitudinality is a defining feature; students are assigned to a consistent community for multiple years, often with vertically integrated activities that include senior students and residents [9].

A national study describes three predominant LC models, which frequently overlap in practice [9]. The professional development model centers on advising, mentoring, and coaching, often linked to assessment processes or longitudinal curricular threads such as clinical skills and professionalism. The well-being and integration model focuses on programmatic wellness initiatives, transition support, and partnerships with student affairs resources to promote help-seeking and community connection. Thirdly, the community and identity model mirrors collegiate house systems, emphasizing belonging, rituals, vertical integration, community service, and peer networks.

Across these models, several common design elements are associated with high-functioning learning communities. Programs that meet regularly and maintain continuity foster trust and timely guidance. Multi-dimensional programming, incorporating seminars, advising, wellness activities, service, and social events, addresses diverse learner needs. Faculty development and recognition are essential for sustainability, ensuring that mentors and coaches are well-prepared and supported. Integration with assessment and student support systems allows LCs to link reflection and feedback with individualized learning plans. Intentional vertical integration, including near-peer mentoring by senior students or residents, provides authentic role modeling and leadership opportunities. Finally, successful programs use data-informed improvement processes, combining participation metrics, advising access, wellness indicators, and learning-environment measures to guide ongoing refinement.

A summary of these common design elements is presented in Table 1.

**Table 1:** Common design elements of high-functioning learning communities

Design element	Purpose/Impact	VUSM example (colleges program)
Regular contact and continuity	Builds trust, supports early identification of needs, enables timely guidance	Weekly College group sessions during pre-clerkship phase; four-year longitudinal assignment to same College and faculty mentors
Multi-dimensional programming	Integrates seminars, advising, wellness, service, and social activities to meet diverse learner needs	Weekly seminars on ethics, leadership, and professional identity formation; Annual College Cup wellness event; advising and near-peer mentoring
Faculty development and recognition	Ensures quality and sustainability of mentoring through training and protected effort	Dedicated FTE for College Mentors; structured annual faculty development curriculum (Table 6)
Integration with assessment and support systems	Connects learning plans, feedback, and wellness resources	Separate portfolio coaching program with access to competency data; LCME-aligned role boundaries; portfolio reviews
Peer mentorship and vertical integration	Fosters role modeling and leadership among senior learners	Second-year near-peer mentors assigned within each College; vertically integrated College groups include M1-M4 students
Data-informed improvement	Uses mixed methods to continuously refine programs	Annual course evaluations (Tables 4 and 5) and faculty development feedback drive iterative curriculum revisions

## Impact of Learning Communities

Learning communities are known to have a broad impact on all participants, as outlined below and summarized in Table 2:

**Table 2:** Comparative models and best practices

LC model [9]	Typical components	Example outcomes and evidence
Professional development	Portfolio/learning plans; longitudinal clinical-skills thread; advising aligned to milestones/EPAs; protected 1:1 coaching	Improved clerkship performance [19]; greater comfort at clerkship start [18]; higher learning-environment/advising ratings [11]; parity/modest gains on exams in integrated models [20]
Well-being/integration	Wellness curriculum; transition supports; pass/fail and contact-hour reforms; embedded referral pathways; near-peer support	Improved mental health/distress with programmatic/curricular changes [13, 14]; lower burnout and better QoL/empathy with higher LC engagement [15]
Community/Identity	Collegiate houses; vertical integration; rituals and co-curriculars; peer networking	Stronger peer relationships [12]; more positive perceptions of support and climate [11]

Evidence map: LC model → components → outcomes (with exemplar citations)

### Learning Environment, Advising, and Mentoring

By design, LCs promote closer student–faculty connections, which support professional identity formation, mentorship, and role modeling [9]. This aligns with classic work on the student–teacher relationship and the “hidden curriculum as process,” the set of implicit values, behaviors, and professional norms conveyed through everyday interactions, that shape physician identity [10]. The strongest empirical signal is improvement in the learning environment and advising access. Multi-institutional studies report higher ratings of support and climate and more reliable mentoring networks [9, 11]. LC participation has also been associated with stronger peer relationships among medical students [12].

### Well-being and Sense of Community

LCs create a dependable social and educational home base. Regular small-group meetings and supportive faculty and peer relationships can reduce isolation, normalize help-seeking, and serve as a conduit to institutional wellness resources [13].

Programmatic wellness interventions and curricular redesigns, including pass/fail grading, streamlined classroom contact hours to reduce student overload, and proactive counseling, have demonstrated improvements in medical student mental health and distress [14]. Within LC contexts specifically, greater engagement has been associated with lower burnout and better quality of life and empathy [15]. Additional program-level evaluations of academic houses, a form of learning communities, reported wellness benefits [16].

In addition to benefits for students, participation in learning communities has been shown to positively influence faculty experience. Wagner and colleagues found that sustained involvement as learning community faculty was associated with higher professional satisfaction, greater sense of connection to students and colleagues, and enhanced meaning in teaching. Faculty reported that longitudinal relationships with learners strengthened their own professional identity and renewed their enthusiasm for medical education [17]. These findings suggest that LCs may contribute to faculty vitality and retention by fostering purpose, community, and engagement within academic medicine.

### Academic and Clinical Skill Development

Early introduction of clinical-skills teaching has been associated with greater student comfort at the start of third-year clerkships [18]. LC-based pre-clerkship clinical-skills programs have also been linked to improved clerkship performance, and reports from integrated LC models show comparable or modestly better examination outcomes than curricula preceding LC implementation [19, 20]. These findings align with broader observations that continuous coaching and feedback, hallmarks of LCs, strengthen learners’ competencies over time [19, 21, 22].

## Case Study: Vanderbilt University School of Medicine (VUSM)

Vanderbilt University School of Medicine (VUSM) employs an integrated, competency-based curriculum known as *Curriculum 2.0*, which emphasizes self-directed learning, longitudinal assessment, and individualized growth across four years. The curriculum is designed to support the development of Master Adaptive Learners through a combination of foundational science, early clinical immersion, and structured reflection [23]. 

At the heart of this curriculum is the College Program, VUSM’s formal learning community structure, launched in 2007 alongside a comprehensive wellness initiative [14]. The Colleges provide longitudinal mentorship, community, and structured reflection that anchor student development throughout training. In 2010–2011, an academic component, *Colloquium*, was added to integrate professional identity formation and ethics into the pre-clerkship curriculum. This weekly, small-group course is led by longitudinal college-affiliated faculty who facilitate reflection and discussion of topics such as ethics, professional identity formation, servant leadership, health equity, and the broader healthcare environment within each College cohort [24].

Complementing the College Program is the Portfolio Coaching Program, a distinct, data-informed model established in 2013 that supports individualized, competency-based reflection and goal setting. Together, these systems create a cohesive framework that balances relationship-centered mentorship with personalized, data-driven guidance.

### Vanderbilt’s Integrated Learner Support Model

- **Four Colleges** provide a stable framework for community and professional development, serving as the foundation for small-group learning, longitudinal mentorship, and vertically integrated relationships among peers, near-peers, and faculty.
- **Weekly College group sessions** during the pre-clerkship phase facilitate discussion of ethics, empathy, leadership, health policy, and professional identity in small cohorts led by two dedicated longitudinal faculty mentors (College Mentors*)* who guide student growth and reflection throughout the four-year curriculum.
- **Faculty preparation tailored to the role** develops College Mentors’ skills in facilitating discussion and guiding reflection. This role is central to Vanderbilt’s broader longitudinal model, and sustained institutional support has enabled the program’s stability for more than a decade.
- **Portfolio-based coaching**, while not part of the College structure, functions as a complementary program that aligns with the school’s competency-based assessment framework. This alignment allows coaching to enhance the reflection and feedback practices cultivated with the Colleges, rather than duplicate them [25].

### The Colleges

Upon matriculation, all incoming students participate in a sorting ceremony that simultaneously assigns them to one of the four Colleges and to a stable small-group cohort of approximately 25 peers, while also revealing their two faculty College Mentors. College Mentors are physician-educators. Each pair of College Mentors is assigned to a single cohort/College and accompanies that cohort longitudinally across all four years of medical school, ensuring relational continuity and deep familiarity with each student’s growth trajectory. Each academic year the College Mentor pair are assigned a new cohort of approximately 25 first-year students to replace the approximate 25 students who graduate.

The cohort size of approximately 25/year was determined through deliberate program design: this number is large enough to sustain a diverse range of perspectives while remaining small enough to foster the close relationships and psychological safety essential for reflective practice and mentorship. Structural factors considered included the ratio of students to faculty mentors, room capacity for small-group facilitation, and the goal of meaningful vertical integration across class years. The cohort size was finalized by the College Program leadership in consultation with the curriculum committee. Students are assigned to Colleges through a process that intentionally balances each cohort across demographic characteristics, including gender and background, to promote diversity within each learning community. Each first-year student is paired with a second-year near-peer mentor from the same College, ensuring relational continuity and a shared community context. Near-peer assignments are made by program leadership with attention to mutual fit and, where possible, shared interests or backgrounds, reinforcing the intentional community design of the Colleges. Students remain in these longitudinal College groups for the duration of their time in medical school.

The Colleges’ design emphasizes intentional team and cohort building beginning with the seven-day Foundations of the Profession course that occurs the first week of medical school. In this course, students meet daily with their College Mentors and College peers to foster relationships, promote community-building, and support early professional identity work as they explore ethical and historical frameworks for understanding the practice and profession of medicine [26]. This continuity extends throughout the pre-clerkship year in weekly sessions, where students further develop reflective and professional skills within their College groups. Additional social and wellness activities, such as College Cup, an annual series of sporting, creative, and intellectual competitions, reinforce belonging, inclusion, and College identity across all four groups.

Early outcomes analyses of Vanderbilt’s Colleges documented improvements in wellness supports and career counseling and described early curricular innovations [14, 27–29].

### Student Curriculum

Within the Colleges, the academic component is designed to maximize student learning related to professional identity formation (PIF). The course helps students build an accurate understanding of the medical profession and develop the skills required to function effectively within the healthcare environment. PIF is addressed as a developmental process encompassing knowledge, skills, and attitudes related to one’s practice and the context in which that practice occurs.

The first-year College-based course runs alongside and complements the first-year preclinical course. It emphasizes reflection, identity, art of medicine, service, leadership, and the broader healthcare environment (Table 3). Servant leadership, a concept central to early sessions, refers to the leadership philosophy in which the primary goal is to serve others—in medicine, this translates to prioritizing patients’ and communities’ needs above personal gain, and modeling professional humility and ethical responsibility. Similarly, the Art of Medicine series focuses on the interpersonal and humanistic dimensions of medicine, complementing the scientific rigor and content of the broader first-year curriculum. Content experts from relevant domains (e.g., ethics, health systems science, population health, leadership) collaborate on curriculum design and deliver foundational knowledge. College Mentors facilitate weekly College-group discussion and reflective exercises aligned to each topic within the course.

**Table 3:** VUSM first-year student learning communities college-based course

Timing	Session title	Session objectives
July-Sept (Servant Leadership)	Emotional Intelligence	Servant leadership in medicine Five EI components Role of self-awareness in leadership
	Time Management	Workload prioritization Minimizing distractions “Urgency addiction” and priorities
	Conflict Management	Recognizing charged communication STATE skills for conversations Thomas-Kilmann conflict modes
Oct-Dec (Art of Medicine)	Reflection in Medicine	Value of reflection Building compassion and self-care Group reflection for identity
	Mindfulness	Mindfulness vs. meditation Role of attention in practice Practical strategies
	Narrative Medicine	Definition and role in care Self-reflective practice Navigating ethics and bias
Jan-Mar (Clinical Ethics)	Practice of Clinical Ethics	Common dilemmas Ethical frameworks and tools Shared decision-making
	Ethics of End-of-Life Care	Challenges in EOL care Surrogate standards Management strategies
	Relationship of Ethics, Law, and Health Policy	Roles of science/stakeholders Privacy and confidentiality Consensus in complex issues
	Ethics in Research	Principles of human research Engaging diverse populations Balancing conflicting principles
Apr-Jun (Metacognition in Clinical Care)	Metacognition and Cognitive Predispositions	Using metacognition to manage predispositions Dual process theory Identifying implicit predispositions
	Preparation for Clinical Clerkships	Creating positive environments Role of empathy in care Managing feedback effectively

Each session begins with a 30-minute context talk by a subject matter expert followed by a 90-minute small group discussion within the College

The Foundations of the Profession course and subsequent weekly sessions were developed through a structured curricular design process involving the College Program leadership, content-area faculty experts, and the school’s Undergraduate Medical Education Committee. Instructional topics were selected based on LCME competency domains, VUSM’s competency framework, and the programmatic goal of supporting professional identity formation throughout training. The curriculum undergoes annual review informed by student evaluation data and faculty feedback, with modifications discussed collaboratively between College leadership, the student curriculum committee and the Undergraduate Medical Education Committee. The trusting environment created within the Colleges supports students’ growth in metacognition and clinical reasoning, ethics and professionalism, healthcare for all, service, and leadership, along with a broader understanding of the healthcare system. Academic sessions are developmentally appropriate across phases of the curriculum and intentionally integrated with other longitudinal courses and basic-science blocks, reinforcing key concepts over time.

Longitudinal evaluation data (Tables 4 and 5) collected from Academic Year (AY) 2016–17 through AY2023–24 demonstrate the sustained strength of the College-based course in supporting student learning and development. In the first-year pre-clerkship course, ratings have remained consistently positive across nearly a decade of data, with recent AY2023–24 results showing high marks for clarity of objectives (mean 3.87/5), quality of content (3.72/5), course leadership (3.83/5), and an overall course rating of 3.75/5. These results highlight the curriculum’s durability in supporting students during their transition into medical school. A transient dip in some ratings observed in AY2022–23, most notably in course leadership satisfaction (mean 3.57 vs. prior-year means of 4.2–4.3), likely reflects students’ concerns regarding the Clinical Ethics portion of the curriculum. The course director worked with faculty from the Center for Biomedical Ethics to create a more practical, clinically focused curriculum delivered by practicing clinicians. Ratings recovered in AY2023–24, consistent with programmatic stabilization. As students advance into their clinical year, College-based course ratings remain strong, with AY2023–24 learners reporting that continued facilitated reflection opportunities with College Mentors and peers help them process clerkship experiences (mean score of 4.38/5). Importantly, across cohorts, the majority of students report drawing on skills and knowledge gained in College-based courses during their clerkships, with 88% of AY2023–24 students indicating that they used these skills “sometimes” or “often.” Taken together, these findings illustrate not only the immediate impact of the course curriculum but also the enduring effectiveness of the Colleges model in equipping students for both preclinical and clinical learning across time. These data are end-of-year course evaluations collected from all students as part of routine course administration by phase leadership and the Undergraduate Medical Education Committee, used here for descriptive program evaluation rather than research.

**Table 4:** First-year student LC college-based course evaluation data

Statement (1 = strongly disagree, 5 = strongly agree)	Mean (Standard Deviation)				
	2019–2020 (*n* = 97)	2020–2021 (*n* = 90)	2021–2022 (*n* = 94)	2022–2023 (*n* = 94)	2023–2024 (*n* = 93)
Quality of Content	3.89 (0.81)	4.02 (0.71)	3.97 (0.90)	3.49 (0.95)	3.72 (0.92)
Clarity of Course Objectives	3.84 (0.88)	3.99 (0.77)	4.00 (0.89)	3.59 (1.03)	3.87 (0.93)
Satisfaction with Course Leadership	4.22 (0.84)	4.28 (0.75)	4.23 (0.82)	3.57 (1.06)	3.83 (1.03)
Overall Course Rating	3.99 (0.82)	4.07 (0.84)	3.96 (0.91)	3.51 (1.00)	3.75 (0.89)

Data presented as mean (SD)

### Faculty Development and Support

The Colleges are sustained by deliberate institutional investment in faculty time and infrastructure. All College Mentors receive dedicated full time equivalent (FTE) support that protects their non-clinical time, ensuring they can reliably facilitate College-group discussions, mentor students, and foster reflective practice. Faculty also participate in a structured development curriculum, which combines internal workshops with external speakers, ensuring ongoing growth in mentoring, coaching, and facilitation skills (Table 6). This financial and educational commitment affirms the value of faculty roles within the curriculum and helps maintain program continuity. Faculty perceptions of the College Mentor role have been examined in national studies: Wagner and colleagues found that sustained learning community faculty involvement was associated with higher professional satisfaction, greater sense of connection, and renewed meaning in teaching [17]. At VUSM, informal faculty feedback gathered has similarly reflected high levels of engagement and purpose among College Mentors. Formal longitudinal assessment of faculty experience represents an important next step, and future work should include systematic collection of faculty survey data to characterize the perceived burden, rewards, and sustainability of this role—particularly at institutions with limited teaching faculty.

**Table 5:** Second-year student LC college-based course evaluation data

Statement	Mean (Standard Deviation)				
	2019–2020 (*n* = 49)	2020–2021 (*n* = 53)	2021–2022 (*n* = 45)	2022–2023 (*n* = 94)	2023–2024 (*n* = 98)
LC helped me process experiences I had on my clerkship (5-point Likert scale: 1 = strongly disagree, 5 = strongly agree)	4.61(0.60)	4.25 (0.84)	4.09 (0.89)	4.30 (0.81)	4.38 (0.72)
I used LC skills on my clerkship (4-point Likert scale: 1 = Often, 4 = Never)	3.29 (0.76)	2.98 (0.79)	2.89 (0.85)	3.05 (0.75)	3.03 (0.77)

Data presented as mean (SD)

**Table 6:** VUSM college mentor professional development curriculum

Timing	Session titles	Session objectives
June	Introduction to Learning Communities	Definition, functions, goals of LC at VUSM Year structure overview
July	Mentor and Facilitation Role in Learning Communities	Roles of mentor and facilitator Best practices in small-group learning
August	Introduction to Professional Identity Formation	Definition of PIF Role of socialization, role models, reflection Key strategies to promote PIF
September	Small Group Facilitation Skills Workshop, Part 1	Facilitator vs. student roles Psychological safety Best practices for sessions
October	Student Support Resources	Common student struggles VUSM resources Supporting students of concern
November	Providing Feedback on Reflective Writing	Goals of written feedback Tools and frameworks Facilitate practice with samples
December	Facilitating Race and Identity Discussions, Part 1	Shared vocabulary Structural racism and bias Tips for effective conversations
January	Small Group Facilitation Skills Workshop, Part 2 (External Consultant)	Advanced facilitation skills Managing group dynamics Long-term cohort strategies
February	Coaching Skills and Techniques	Coaching mindset Asking effective questions Tools and brief peer coaching
March	Using Non-Cognitive Attributes for Assessment	Definitions Challenges in assessment Observable practice activities for feedback
April	Facilitating Race and Identity Discussions, Part 2	History of Nashville Racial identity frameworks Learner applications
May	Year in Review	Reflect on experiences Gather rapid-cycle feedback Plan for next year

Each session runs for 120 min and follows an interactive format, with mentor participation and engagement facilitated by course leadership or an external consultant

### Complementary Portfolio Coaching Program

The Portfolio Coaching Program, established in 2013, operates separately from the Learning Community structure but complements its goals by providing individualized, competency-based guidance. Each student is paired early in training with a coach who uses portfolio data and competency milestones to promote reflection, self-assessment, and longitudinal goal setting.

College Mentors and Portfolio Coaches serve distinct yet coordinated functions within Vanderbilt’s learner-support ecosystem. College Mentors, who are assessment-blind, cultivate belonging, reflection, and professional identity formation within the Colleges, fulfilling LCME Standard 11.1 (Academic Advising and Career Counseling) in a relational, non-evaluative capacity. Portfolio Coaches, who have access to assessment data, help students synthesize feedback and monitor progress over time, fulfilling Standard 9.7 (Formative Assessment and Feedback). Faculty serve exclusively in one role. College Mentors do not have access to summative assessment outcomes, grades, or performance data, and they are not involved in academic advising decisions tied to academic standing or remediation; Coaches do not participate in facilitated College group sessions. This structural firewall is reinforced through faculty onboarding, annual development sessions, and program policy, and both roles receive protected effort and development support to ensure quality and sustainability. Together, the Colleges and Portfolio Coaching Program form a cohesive system that integrates relationship-centered mentorship with structured, competency-based coaching [30].

#### Notable Strengths

- **Longevity**: Nearly two decades of continuous operation provide a strong foundation for outcomes tracking and program refinement.
- **Breadth of integration**: The combination of wellness initiatives and structured curricular content distinguishes Vanderbilt’s model from those focused solely on advising or coursework.
- **Relational continuity**: Stable faculty mentors and longitudinal small groups foster belonging, trust, and consistent professional guidance across all four years.
- **Faculty investment**: Protected time and structured development for mentors ensure high-quality facilitation and program sustainability.
- **Professional identity formation**: The Colleges’ reflective curriculum and mentor-guided discussions make identity development an intentional and visible component of medical training.
- **Complementary ecosystem**: A separate, competency-based coaching program reinforces feedback and reflection practices initiated within the Colleges while preserving clear boundaries between advising and assessment.

## Implementation Guide (With Pitfalls)

Based on previously published program descriptions, available research data, and our own institutional experiences, we offer a comprehensive guide for those interested in implementing or enhancing learning community programs in undergraduate medical education. We aim to create a stepwise guide for new or existing programs to foster learning community success while avoiding common barriers seen both at our institution and nationally.

1. Define purpose and outcomes
  Clarify goals (identity formation, wellness, advising, curriculum delivery) and map them to measurable indicators. At VUSM, the Colleges were designed with explicit goals around professional identity formation, belonging, and wellness, measured through annual course evaluations.
  *Pitfall: If goals are vague*,* outcomes become difficult to measure and assess.*
2. Select the structural model
  Choose the balance of mentoring/advising/coaching and curricular delivery; define cohort size, vertical integration, and frequency of meetings. VUSM selected a model integrating formal curriculum delivery with longitudinal mentorship, organizing students into four Colleges of approximately 25 students per cohort, meeting weekly during the pre-clerkship year, with same-College assignment across all four years.
  *Pitfall: A rigid or misaligned model may not fit institutional context.*
3. Staff and prepare faculty
  Recruit committed faculty; provide training in the requisite skills for their new role (coaching, feedback, and assessment); align effort with recognition and workload policies.
  *Pitfall: Faculty turnover and burnout can occur if roles are under-recognized*,* under-supported*,* or overloaded.*
4. Integrate with assessment
  Implement learner portfolios; align LC touchpoints with milestones/EPAs; schedule periodic portfolio reviews.
  *Pitfall: If LCs are seen as an “add-on*,*” their value diminishes.*
5. Design the LC curriculum
  Create a longitudinal thread (e.g., clinical skills, ethics, leadership) with session guides, cases, and reflective prompts. At VUSM, the College-based curriculum thread addresses professional identity, ethics, servant leadership, and healthcare for all in Year 1, extending into facilitated clerkship reflection in Years 3–4 (see Table 3).
  *Pitfall: Without clear scope*,* sessions may drift into purely social events or overly didactic lectures.*
6. Embed wellness supports
  Include structured wellness activities and referral pathways; normalize help-seeking and peer support.
  *Pitfall: Treating wellness as optional or invisible undermines its impact.*
7. Protect time and space
  Build LC sessions and 1:1 meetings into the master schedule; ensure rooms/resources support small-group work.
  *Pitfall: Inconsistent cadence or conflicts with other curriculum erode continuity.*
8. Measure and iterate
  Track participation, advising access, wellness indicators, portfolio engagement, and learning-environment measures; use findings to drive continuous improvement. VUSM uses annual course evaluations (Tables 4 and 5), faculty development feedback, and periodic programmatic reviews to inform iterative improvements, including curriculum updates and faculty coaching enhancements.
  *Pitfall: Collecting data without acting on it reduces credibility and momentum.*

## Future Directions and Research Priorities

Future work on learning communities should extend beyond student perceptions to evaluate outcomes such as clinical performance, academic progression, professionalism milestones, and residency placement. Multi-institutional studies are particularly needed to generate generalizable evidence. Equally important is investigating the mechanisms of impact—specifically how coaching, a sense of belonging, and structured reflection mediate student development. Research should also prioritize inclusion and belonging by identifying and disseminating best practices for fostering inclusive mentorship across diverse learner populations. Longitudinal follow-up of graduates who participated in LC programs is also warranted; with outcomes data now extending back to 2016–17 at VUSM, future studies should survey alumni in residency and practice to assess how LC participation shaped their professional identity, well-being, and career trajectory. Additionally, programs should systematically document and share how student feedback has been incorporated into iterative curriculum improvement—an area that has informed ongoing refinements at VUSM but merits formal study and broader dissemination.

## Conclusion

Well-designed learning communities foster belonging, mentorship, and professional identity formation through consistent relationships, reflective dialogue, and coherent curricular threads that span all phases of training. By strengthening the learning environment and cultivating longitudinal connections among students and faculty, LCs provide the foundation on which professional growth occurs. At Vanderbilt, this structure is further enhanced by individualized portfolio coaching, which complements, rather than replaces, the reflection and feedback practices embedded within the Colleges. Together, these integrated systems create a supportive, data-informed ecosystem that promotes adaptive expertise, well-being, and a deepened sense of professional purpose across the continuum of medical education.
